# Relationship Between Alcohol Drinking Pattern and Risk of Proteinuria: The Kansai Healthcare Study

**DOI:** 10.2188/jea.JE20150158

**Published:** 2016-09-05

**Authors:** Shinichiro Uehara, Tomoshige Hayashi, Kyoko Kogawa Sato, Shigeki Kinuhata, Mikiko Shibata, Keiko Oue, Hiroshi Kambe, Kunihiko Hashimoto

**Affiliations:** 1 Department of Preventive Medicine and Environmental Health, Osaka City University Graduate School of Medicine, Osaka, Japan; 1 大阪市立大学大学院医学研究科 産業医学; 2 Kansai Health Administration Center, Nippon Telegraph and Telephone West Corporation, Osaka, Japan; 2 西日本電信電話株式会社 関西健康管理センタ

**Keywords:** alcohol, drinking pattern, proteinuria, chronic kidney disease, prospective study, アルコール, 飲酒パターン, 蛋白尿, 慢性腎臓病, 前向きコホート研究

## Abstract

**Background:**

Moderate alcohol consumption has been reported to be associated with a decreased risk of cardiometabolic diseases. Whether drinking pattern is associated with the risk of proteinuria is unknown.

**Methods:**

Study subjects were 9154 non-diabetic Japanese men aged 40–55 years, with an estimated glomerular filtration rate ≥60 mL/min/1.73 m^2^, no proteinuria, and no use of antihypertensive medications at entry. Data on alcohol consumption were obtained by questionnaire. We defined “consecutive proteinuria” as proteinuria detected twice consecutively as 1+ or higher on urine dipstick at annual examinations.

**Results:**

During the 81 147 person-years follow-up period, 385 subjects developed consecutive proteinuria. For subjects who reported drinking 4–7 days per week, alcohol consumption of 0.1–23.0 g ethanol/drinking day was significantly associated with a decreased risk of consecutive proteinuria (hazard ratio [HR] 0.54; 95% confidence interval [CI], 0.36–0.80) compared with non-drinkers. However, alcohol consumption of ≥69.1 g ethanol/drinking day was significantly associated with an increased risk of consecutive proteinuria (HR 1.78; 95% CI, 1.01–3.14). For subjects who reported drinking 1–3 days per week, alcohol consumption of 0.1–23.0 g ethanol/drinking day was associated with a decreased risk of consecutive proteinuria (HR 0.76; 95% CI, 0.51–1.12), and alcohol consumption of ≥69.1 g ethanol/drinking day was associated with an increased risk of consecutive proteinuria (HR 1.58; 95% CI, 0.72–3.46), but these associations did not reach statistical significance.

**Conclusions:**

Men with frequent alcohol consumption of 0.1–23.0 g ethanol/drinking day had the lowest risk of consecutive proteinuria, while those with frequent alcohol consumption of ≥69.1 g ethanol/drinking day had an increased risk of consecutive proteinuria.

## Abstract (ja)

**【背景】:**

適度な飲酒は心血管疾患、代謝疾患の発症リスクを低下させるという報告がある。飲酒パターンが蛋白尿の発症リスクとの関係があるかどうかについては不明である。

**【方法】:**

対象は、糖尿病のない、推算糸球体濾過量が60 mL/min/1.73 m^2^以上で、蛋白尿を認めず、高血圧の治療を受けていない、40–55歳の日本人男性9154人である。問診票によりアルコール週当たりの飲酒日数と飲酒日のアルコール摂取量を得た。発症は、毎年の試験紙法による尿検査で1+以上を蛋白尿とし2年連続認めたものを「連続蛋白尿」と定義した。

**【結果】:**

81147人年の経過で385人の連続蛋白尿の発症を認めた。多変量解析の結果、週当たりの飲酒日が4–7日で飲酒日に0.1–23.0 gエタノール摂取者は、非飲酒者と比べて、連続蛋白尿発症リスクの低下を認めた (多変量補正後のハザード比0.54 (95％信頼区間0.36–0.80)）。一方で同頻度で飲酒日に69.1 gエタノール以上の摂取者は有意に連続蛋白尿発症リスクが高かった (1.78 (1.01–3.14))。飲酒日が1-3日に0.1–23.0 gエタノール摂取者は連続蛋白尿の発症リスクの低下を認めた（0.76（0.51-1.12））。また、同頻度で飲酒日に69.1 gエタノール以上の摂取者は連続蛋白尿発症リスクが高かった (1.58（0.72–3.46）)が統計学的な有意差を認めなかった。

**【結論】:**

飲酒日のアルコール摂取量が0.1–23.0 gエタノールの頻回飲酒者は最も蛋白尿の発症リスクが低かった。一方、頻回の69.1 gエタノール以上の摂取者は蛋白尿の発症リスクが高かった。

## INTRODUCTION

Chronic kidney disease (CKD) is a major public health problem worldwide. Proteinuria has been reported to be associated with the risk of end-stage renal disease and cardiovascular diseases.^1^^,^^2^ However, the factors associated with the risk of future proteinuria have not been fully examined.

Many previous epidemiological studies have reported associations between moderate alcohol consumption and a lower risk of type 2 diabetes^3^ or cardiovascular disease.^4^ Only two prospective studies are available that relate average daily alcohol consumption to the risk of future proteinuria or albuminuria.^5^^,^^6^ However, the results of these studies were inconclusive. Because both studies assessed average daily alcohol consumption, it was impossible to distinguish subjects who consume a large amount of alcohol on a few days of the week from those with the same weekly alcohol consumption who were regular light drinkers. In addition, these studies did not examine the association between drinking pattern, which took into account both the weekly frequency of drinking and the quantity of alcohol consumption per drinking day, and the risk of future proteinuria.

Previous epidemiological studies have reported that drinking pattern has a major influence on the risk of cardiometabolic diseases, including coronary heart disease, type 2 diabetes, ischemic stroke, and cancer.^7^^–^^12^ However, to our knowledge, no epidemiological study has examined the association between drinking pattern and the risk of future proteinuria.

Therefore, we prospectively examined the association between drinking pattern, which was defined as a combination of the alcohol quantity per drinking day and the number of days of drinking per week, and the risk of proteinuria.

## METHODS

### Study design and subjects

The Kansai Healthcare Study is an ongoing cohort investigation designed to clarify the risk factors for chronic diseases.^13^^,^^14^ Between April 2000 and March 2001, 12 647 male workers of a company in the Kansai region of Japan, who were aged 40–55 years at entry and considered to be involved in sedentary jobs, were enrolled in this study. All employees of this company aged 40 years or older have undergone detailed annual medical check-ups. This study was approved by the Human Subjects Review Committee at Osaka City University.

For the current analysis, we included 10 019 subjects at entry who had an estimated glomerular filtration rate (eGFR) ≥60 mL/min/1.73 m^2^, no proteinuria, no use of antihypertensive medications, a fasting plasma glucose <126 mg/dL, and who were not taking hypoglycemic medications or insulin. We excluded 320 men who did not undergo medical check-ups during the follow-up period and 545 men with missing covariate information. Thus, the final study population consisted of 9154 men.

### Data collection and measurements

The clinical examination consisted of a medical history, physical examination, anthropometric measurements, self-administered questionnaires on lifestyle factors, dipstick urinalysis, and measurement of blood pressure, fasting plasma glucose, serum creatinine, total cholesterol, triglycerides (TG), and high-density lipoprotein cholesterol (HDL-C). Low-density lipoprotein cholesterol (LDL-C) levels were calculated using the Friedewald formula in subjects with TG <400 mg/dL.^15^ Trained nurses took all measurements. Urine samples were collected as clean-catch, mid-stream, and random urine specimens. The results of dipstick urinalysis were classified as negative, ±, 1+, 2+, 3+, or 4+, with negative or ± being regarded as normal. Blood samples were drawn after a 12-h overnight fast. Serum creatinine was mainly measured using an enzymatic method using a Hitachi 7350 automatic chemistry analyzer (Hitachi Ltd., Tokyo, Japan). Serum creatinine was measured using the Jaffe method in 1612 subjects at the baseline examination, so the Jaffe values were recalibrated to correspond to enzymatic values using the following equation developed by the analytical laboratory: serum creatinine (mg/dL, enzymatic method) = 1.02 × serum creatinine (mg/dL, Jaffe method) − 0.25 (*r* = 0.9996). Then, we calculated eGFR using the Modification of Diet in Renal Disease equation for Japanese persons, which has been validated by the standard inulin clearance technique: eGFR = 194 × age^−0.287^ × serum creatinine^−1.094^ (mg/dL, enzymatic method).^16^ Blood pressure was measured in the sitting position with a standard automated sphygmomanometer (BP-203RV; Omron Colin, Tokyo, Japan, or Udex-super; ELK Corp., Osaka, Japan) after the subject had rested for approximately 5 minutes in a quiet room. Body mass index (BMI) was calculated as the weight in kilograms divided by the square of the height in meters. Hypertension was defined as systolic blood pressure ≥140 mm Hg or diastolic blood pressure ≥90 mm Hg.^17^ Dyslipidemia was defined as one or more of the following components: 1) TG ≥150 mg/dL, 2) HDL-C <40 mg/dL, 3) LDL-C ≥140 mg/dL, or 4) use of oral lipid-lowering medications.^18^

The questionnaire about alcohol consumption assessed the weekly frequency of drinking alcohol (the number of days per week) and the quantity of alcohol per drinking day according to Japanese standard drinks. One Japanese standard drink is 23 g of ethanol. Average daily alcohol consumption was calculated as follows: (alcohol consumption per drinking day) × (the number of drinking days per week)/7. Based on their average daily alcohol consumption, the subjects were categorized as follows: non-drinkers, 0.1–23.0 g ethanol per day, 23.1–46.0 g ethanol per day, 46.1–69.0 g ethanol per day, and ≥69.1 g ethanol per day. Based on the number of drinking days per week, the subjects were also categorized as follows: non-drinkers, drinking on 1–3 days per week, or drinking on 4–7 days per week. Based on the alcohol consumption per drinking day, subjects were categorized as follows: non-drinkers, 0.1–23.0 g ethanol per drinking day, 23.1–46.0 g ethanol per drinking day, 46.1–69.0 g ethanol per drinking day, and ≥69.1 g ethanol per drinking day. Drinking patterns were then determined by combining the alcohol consumption per drinking day with the number of drinking days per week.

To assess leisure-time physical activity, a single question was used with three possible answers: rarely, sometimes, or regularly (at least once a week). Subjects were classified as engaging in leisure-time physical activity at least once weekly or less than once weekly.^13^ Regarding smoking habits, subjects were classified as current smokers, past smokers, or non-smokers.

### Outcomes

Proteinuria was defined as 1+ or higher (30 mg/dL or higher) on urine dipstick examination at the annual medical check-up. We used two definition of proteinuria: “any proteinuria” included subjects in whom proteinuria was detected at least once during the follow-up period; “consecutive proteinuria” included subjects in whom proteinuria was detected twice consecutively during the follow-up, to exclude chance proteinuria as much as possible.

### Statistical analysis

We used the Cox proportional hazards model to investigate the risk of future any proteinuria or consecutive proteinuria in relation to the average daily alcohol consumption or the drinking pattern. We adjusted the multivariate models for age, BMI categories (<18.5, 18.5–24.9, 25.0–29.9, and ≥30.0 kg/m^2^), smoking habits (non-smokers, past smokers, and current smokers), regular leisure-time physical activity (yes/no), hypertension (yes/no), fasting plasma glucose, and eGFR at baseline. Follow-up of each subject was continued until an outcome was detected or until the 11-year follow-up examination (between April 1, 2011 and March 31, 2012), whichever came first. In all multivariate models, nonlinear effects of continuous independent variables were evaluated by plotting the regression coefficients against the variables.^19^ Nonlinear effects of continuous independent variables were also evaluated using quadratic, square root, and log transformations, which were tested in the Cox proportional hazards models to determine whether these transformations improved the linear fit. Of all continuous independent variables assessed in all multivariate models, only BMI did not fulfill the linearity assumption; therefore, we fitted models using BMI as a categorical variable according to the World Health Organization classification of obesity: <18.5, 18.5–24.9, 25.0–29.9, and ≥30.0 kg/m^2^.^20^ The proportional hazards assumption was confirmed by the insertion of time-dependent covariates or by the Schoenfeld residuals plot and Schoenfeld residuals test.^21^ All independent variables met the assumption in all models. The presence of effect modification was tested by the insertion of a first-order interaction term into appropriate regression models. We examined the first-order interaction in all models between average daily alcohol consumption or drinking pattern and the other variables. None of these interactions were statistically significant. Multicollinearity was assessed using the variance inflation factor.^22^ There was no evidence of multicollinearity. We checked for outliers by plotting the likelihood displacement values and LMAX values of all independent variables.^23^ Outliers were not detected in any of our models. We calculated the 95% confidence interval for each hazard ratio. All *P* values were two-tailed and considered statistically significant if the values were less than 0.05. Statistical analyses were performed using Stata MP, version 13.0 (StataCorp., College Station, TX, USA) and PASW Statistics 19.0 (SPSS Inc., Chicago, IL, USA).

## RESULTS

### Baseline characteristics

The baseline characteristics of the subjects by average daily alcohol consumption are summarized in Table 1. Subjects who consumed higher levels of alcohol were likely to have higher systolic and diastolic blood pressure, eGFR, TG, and HDL-C, and lower LDL-C than those who consumed less alcohol. They were also more likely to have hypertension and to be current smokers.

**Table 1.** Baseline characteristics of the subjects stratified according to average daily alcohol consumption

	Total	Average daily alcohol consumption^a^				

		Non-drinkers	0.1–23.0 g ethanol/day	23.1–46.0 g ethanol/day	46.1–69.0 g ethanol/day	≥69.1 g ethanol/day
Number	9154	1397	3929	2909	816	103
Age, years	48.2 (4.2)	48.8 (4.2)	47.9 (4.2)	48.3 (4.1)	48.0 (4.2)	47.2 (4.1)
Body mass index, kg/m^2^	23.2 (2.8)	23.1 (3.1)	23.3 (2.8)	23.3 (2.8)	23.2 (2.9)	23.3 (3.3)
Systolic blood pressure, mm Hg	127.5 (17.6)	123.3 (17.4)	126.0 (17.1)	130.0 (17.5)	131.6 (18.2)	134.8 (19.0)
Diastolic blood pressure, mm Hg	79.5 (11.8)	76.2 (11.4)	78.8 (11.7)	81.3 (11.6)	81.6 (11.6)	83.4 (12.2)
Hypertension, %^b^	27.9	19.5	25.2	32.7	35.2	47.6
Total cholesterol, mg/dL	204.7 (33.1)	207.8 (33.6)	205.1 (32.2)	203.6 (33.5)	202.7 (34.2)	198.3 (36.5)
Triglycerides, mg/dL	138.7 (110.6)	131.2 (83.6)	129.4 (89.1)	145.5 (130.6)	167.4 (148.8)	174.1 (137.2)
High-density lipoprotein cholesterol, mg/dL	56.6 (14.9)	50.5 (12.3)	55.2 (14.0)	59.6 (15.1)	62.2 (17.1)	62.0 (15.8)
Low-density lipoprotein cholesterol, mg/dL^c^	121.8 (31.0)	131.5 (30.5)	124.8 (29.6)	116.6 (30.7)	110.5 (32.0)	102.8 (34.4)
Dyslipidemia, %^c,d^	49.1	57.1	49.3	45.9	45.6	50.5
Fasting plasma glucose, mg/dL	97.2 (9.2)	95.8 (8.9)	97.0 (9.0)	97.8 (9.4)	98.4 (9.4)	98.7 (8.4)
Estimated glomerular filtration rate, mL/min/1.73 m^2^	84.7 (14.0)	83.8 (14.5)	83.9 (14.0)	85.7 (13.7)	85.9 (13.3)	88.9 (13.6)
Smoking habits						
Non-smokers, %	21.3	23.5	26.4	17.2	9.8	8.7
Past smokers, %	21.7	20.5	22.4	22.3	18.4	19.4
Current smokers, %	57.0	56.0	51.2	60.5	71.8	71.9
Regular leisure-time physical activity, %	17.9	13.9	18.2	19.2	18.5	15.5

Data are shown as the % or mean (standard deviation).

^a^Average daily alcohol consumption was calculated as ([alcohol consumption per drinking day] × [the number of drinking days per week]/7). One Japanese standard drink is 23 g of ethanol.

^b^Hypertension was defined as systolic blood pressure ≥140 mm Hg or diastolic blood pressure ≥90 mm Hg.

^c^Low-density lipoprotein cholesterol levels were calculated by the Friedewald formula for subjects with triglyceride levels <400 mg/dL. We excluded 213 subjects with triglyceride levels ≥400 mg/dL.

^d^Dyslipidemia was defined as triglycerides ≥150 mg/dL, high-density lipoprotein cholesterol <40 mg/dL, low-density lipoprotein cholesterol ≥140 mg/dL, or use of oral lipid-lowering medications.

### Average daily alcohol consumption and the risk of any proteinuria

We examined the association between average daily alcohol consumption and the risk of any proteinuria. During the 73 159 person-years of follow-up, 1910 subjects developed any proteinuria. The risks of any proteinuria in relation to average daily alcohol consumption are shown in Table 2. Subjects who had consumed 0.1–23.0 g ethanol/day and 23.1–46.0 g ethanol/day had a lower risk of any proteinuria than non-drinkers after adjustment for age, BMI (<18.5, 18.5–24.9, 25.0–29.9, and ≥30.0 kg/m^2^), smoking habits (non-smokers, past smokers, and current smokers), regular leisure-time physical activity (yes/no), hypertension (yes/no), fasting plasma glucose, and eGFR. Alcohol consumption of 46.1–69.0 g ethanol/day and of ≥69.1 g ethanol/day were not associated with the risk of any proteinuria.

**Table 2.** Incidence rates and hazard ratios for any proteinuria^a^ according to average daily alcohol consumption

Average daily alcohol consumption^b^	Incidence rate^c^ (cases/person-years)	Crude hazard ratio (95% CI)	*P*	Multiple-adjusted hazard ratio (95% CI)^d^	*P*
Non-drinkers	29.1 (317/10 903)	1.00 (reference)		1.00 (reference)	
0.1–23.0 g ethanol/day	22.9 (739/32 337)	0.79 (0.69–0.90)	<0.001	0.79 (0.69–0.90)	<0.001
23.1–46.0 g ethanol/day	26.5 (613/23 092)	0.91 (0.80–1.05)	0.188	0.86 (0.75–0.98)	0.028
46.1–69.0 g ethanol/day	34.1 (207/6070)	1.17 (0.98–1.40)	0.074	1.03 (0.86–1.23)	0.721
≥69.1 g ethanol/day	44.9 (34/757)	1.55 (1.09–2.20)	0.016	1.31 (0.92–1.87)	0.139

CI, confidence interval.

^a^Any proteinuria was defined if proteinuria was detected at least once during the follow-up period.

^b^Average daily alcohol consumption was calculated as ([alcohol consumption per drinking day] × [the number of drinking days per week]/7).

^c^Incidence rates are expressed as the incidence per 1000 person-years.

^d^Adjusted for age, body mass index categories (<18.5, 18.5–24.9, 25.0–29.9, and ≥30.0), smoking habits (non-smokers, past smokers, and current smokers), regular leisure-time physical activity (yes/no), hypertension (yes/no), fasting plasma glucose, and estimated glomerular filtration rate.

### Drinking pattern and the risk of any proteinuria

We next examined the joint association of the number of drinking days per week and the alcohol consumption per drinking day with the risk of any proteinuria because both the weekly number of drinking days and the alcohol consumption per drinking day could not be included in the same model due to multicollinearity (Table 3). The subjects were categorized into 9 drinking pattern groups by combining the number of drinking days per week (non-drinkers, 1–3 days per week, and 4–7 days per week) with the alcohol consumption per drinking day (0.1–23.0, 23.1–46.0, 46.1–69.0, and ≥69.1 g ethanol/drinking day). This analysis revealed that drinking pattern was associated with risk of any proteinuria. Subjects who consumed 0.1–23.0 g ethanol/drinking day and 23.1–46.0 g ethanol/drinking day on 4–7 days per week had a significantly lower risk of any proteinuria than non-drinkers after adjustment for covariates. Subjects who consumed 0.1–23.0 g ethanol/drinking day on 1–3 days per week also had a significantly lower risk of any proteinuria. In contrast, alcohol consumption of ≥69.1 g ethanol/drinking day was significantly associated with an increased risk of any proteinuria, regardless of the weekly number of drinking days.

**Table 3.** Incidence rates and hazard ratios for any proteinuria^a^ according to the drinking pattern

Drinking pattern	Incidence rate^b^ (cases/person-years)	Crude hazard ratio (95% CI)	*P*	Multiple-adjusted hazard ratio (95% CI)^c^	*P*
Non-drinkers	29.1 (317/10 903)	1.00 (reference)		1.00 (reference)	
1–3 drinking days/week					
0.1–23.0 g ethanol/drinking day	23.9 (237/9934)	0.82 (0.69–0.97)	0.022	0.84 (0.71–1.00)	0.045
23.1–46.0 g ethanol/drinking day	27.1 (224/8272)	0.93 (0.79–1.11)	0.420	0.89 (0.75–1.06)	0.199
46.1–69.0 g ethanol/drinking day	34.2 (95/2780)	1.18 (0.93–1.48)	0.168	1.07 (0.85–1.35)	0.561
≥69.1 g ethanol/drinking day	54.4 (30/552)	1.86 (1.28–2.70)	0.001	1.71 (1.17–2.49)	0.005
4–7 drinking days/week					
0.1–23.0 g ethanol/drinking day	19.2 (256/13 355)	0.66 (0.56–0.78)	<0.001	0.66 (0.56–0.78)	<0.001
23.1–46.0 g ethanol/drinking day	24.8 (510/20 535)	0.85 (0.74–0.98)	0.028	0.80 (0.70–0.93)	0.003
46.1–69.0 g ethanol/drinking day	33.3 (194/5818)	1.15 (0.96–1.37)	0.133	1.01 (0.84–1.21)	0.941
≥69.1 g ethanol/drinking day	46.5 (47/1010)	1.60 (1.18–2.18)	0.003	1.38 (1.02–1.88)	0.040

CI, confidence interval.

^a^Any proteinuria was defined if proteinuria was detected at least once during the follow-up period.

^b^Incidence rates are expressed as the incidence per 1000 person-years.

^c^Adjusted for age, body mass index categories (<18.5, 18.5–24.9, 25.0–29.9, and ≥30.0), smoking habits (non-smokers, past smokers, and current smokers), regular leisure-time physical activity (yes/no), hypertension (yes/no), fasting plasma glucose, and estimated glomerular filtration rate.

### Drinking pattern and the risk of consecutive proteinuria

We examined the association between drinking pattern and risk of consecutive proteinuria, which was defined as proteinuria detected twice consecutively during the follow-up period, to exclude proteinuria detected by chance (Table 4). During the 81 147 person-years of follow-up, 385 subjects had consecutive proteinuria. The associations between drinking pattern and risk of consecutive proteinuria tended to be similar to those between drinking pattern and risk of any proteinuria presented above (Table 3). Compared with non-drinkers, subjects who consumed 0.1–23.0 g ethanol/drinking day on 4–7 days per week had a significantly lower risk of consecutive proteinuria. Those who consumed 23.1–46.0 g ethanol/drinking day on 4–7 days per week also had a lower risk of consecutive proteinuria than non-drinkers, but this association did not reach statistical significance. Subjects who consumed ≥69.1 g ethanol/drinking day had an increased risk of developing consecutive proteinuria, regardless of the number of drinking days per week, although this association was only statistically significant for subjects who consumed ≥69.1 g ethanol/drinking day on 4–7 days per week, and not for those with the same alcohol consumption per drinking day on 1–3 days per week.

**Table 4.** Incidence rates and hazard ratios for consecutive proteinuria^a^ according to the drinking pattern

Drinking pattern	Incidence rate^b^ (cases/person-years)	Crude hazard ratio (95% CI)	*P*	Multiple-adjusted hazard ratio (95% CI)^c^	*P*
Non-drinkers	5.4 (66/12 162)	1.00 (reference)		1.00 (reference)	
1–3 drinking days/week					
0.1–23.0 g ethanol/drinking day	3.8 (42/10 969)	0.71 (0.48–1.04)	0.079	0.76 (0.51–1.12)	0.160
23.1–46.0 g ethanol/drinking day	5.8 (53/9193)	1.06 (0.74–1.53)	0.737	1.01 (0.70–1.45)	0.977
46.1–69.0 g ethanol/drinking day	4.3 (14/3225)	0.80 (0.45–1.42)	0.442	0.70 (0.39–1.25)	0.224
≥69.1 g ethanol/drinking day	10.3 (7/677)	1.89 (0.87–4.12)	0.109	1.58 (0.72–3.46)	0.252
4–7 drinking days/week					
0.1–23.0 g ethanol/drinking day	2.8 (41/14 465)	0.52 (0.35–0.77)	0.001	0.54 (0.36–0.80)	0.002
23.1–46.0 g ethanol/drinking day	4.7 (107/22 618)	0.87 (0.64–1.19)	0.382	0.79 (0.58–1.08)	0.137
46.1–69.0 g ethanol/drinking day	6.0 (40/6634)	1.11 (0.75–1.65)	0.596	0.90 (0.61–1.35)	0.617
≥69.1 g ethanol/drinking day	12.5 (15/1204)	2.30 (1.31–4.03)	0.004	1.78 (1.01–3.14)	0.047

CI, confidence interval.

^a^Consecutive proteinuria was defined if proteinuria was detected twice consecutively during the follow-up.

^b^Incidence rates are expressed as the incidence per 1000 person-years.

^c^Adjusted for age, body mass index categories (<18.5, 18.5–24.9, 25.0–29.9, and ≥30.0), smoking habits (non-smokers, past smokers, and current smokers), regular leisure-time physical activity (yes/no), hypertension (yes/no), fasting plasma glucose, and estimated glomerular filtration rate.

### Further analysis

After adjustment for dyslipidemia (yes/no) in addition to the covariates shown in Table 2, Table 3, and Table 4, similar results for each association were obtained (data not shown). We also adjusted the models in Table 2, Table 3, and Table 4 for the mean values of fasting plasma glucose, eGFR, and systolic or diastolic blood pressure during the follow-up period, in addition to age, BMI, smoking habits, and regular leisure-time physical activity at baseline. However, we obtained similar findings to those displayed in Table 2, Table 3, and Table 4 (data not shown).

## DISCUSSION

This prospective investigation demonstrated that, for subjects who consumed alcohol 4–7 days per week, alcohol consumption of 0.1–23.0 g ethanol/drinking day was significantly associated with a decreased risk of future consecutive proteinuria, but alcohol consumption of ≥69.1 g ethanol/drinking day was significantly associated with an increased risk of future consecutive proteinuria. As for subjects who drank alcohol 1–3 days per week, similar results for each association were obtained, but these associations did not reach statistical significance. These associations were independent of age, BMI, smoking habits, regular leisure-time physical activity, hypertension, fasting plasma glucose, and eGFR at baseline.

Only two previous prospective studies have reported the risk of proteinuria or albuminuria in association with average daily alcohol consumption.^5^^,^^6^ Yamagata et al reported that an average daily alcohol consumption ≤20 g of ethanol was associated with a decreased risk of future proteinuria in Japanese men and women compared with non-drinkers, while consumption of >20 g of ethanol was not associated with risk of future proteinuria.^5^ Their finding that light average daily alcohol consumption had a protective effect against proteinuria was consistent with our results, but they did not examine the association between heavy alcohol consumption and the risk of proteinuria. On the other hand, White et al showed in the AusDiab study that, in men under 65 years old, average daily alcohol consumption ≥30 g of ethanol was associated with an increased risk of future albuminuria compared with an average daily alcohol consumption of <10 g of ethanol.^6^ Differences in the categories used for multivariate analysis, exclusion criteria, age distribution, and ethnicity may explain the inconclusive association. Furthermore, neither of the previous studies addressed the association between drinking pattern and proteinuria. Thus, our investigation is the first prospective study to evaluate the association between drinking pattern and risk of future proteinuria.

One of the strengths of this study was that we examined the association between drinking pattern and risk of proteinuria based on two definitions of proteinuria: 1) any proteinuria detected during the follow-up, regardless of the number of times; and 2) consecutive proteinuria, in which proteinuria was detected twice consecutively during the follow-up, to exclude subjects with proteinuria detected by chance. Previous studies on the association between alcohol consumption and the risk of proteinuria or albuminuria have not used the latter definition of proteinuria.^5^^,^^6^

We previously investigated the association between drinking pattern and risk of CKD, which was defined as eGFR <60 mL/min/1.73 m^2^.^24^ We found that subjects who had an alcohol consumption of 23.1–46.0 or 46.1–69.0 g ethanol/drinking day on 4–7 days per week showed a significantly lower risk of developing CKD compared with non-drinkers. However, alcohol consumption of ≥69.1 g ethanol/drinking day was not associated with risk of CKD, regardless of the number of drinking days per week.

The mechanism of the association between alcohol consumption and development of proteinuria has not been examined in detail. Integrity of the glomerular barrier undoubtedly has the most major influence on the development of proteinuria. Among the components of the glomerular filtration barrier, the role of podocytes has attracted attention. An animal study showed that podocytes were insulin-sensitive and that their insulin sensitivity was important in maintaining the integrity of the glomerular filtration barrier.^25^ Furthermore, light to moderate alcohol consumption was reported to increase insulin sensitivity.^26^ Therefore, alcohol consumption might have an influence on the incidence of proteinuria. On the other hand, in the present study, alcohol consumption of ≥69.1 g ethanol/drinking day was associated with an increased risk of proteinuria. Previous epidemiological studies have reported that higher alcohol consumption was associated with an increased risk of future hypertension,^27^ and hypertension or higher blood pressure has been reported to be associated with an increased risk of proteinuria.^5^^,^^28^ Although adjustment for hypertension did not remove the significant association between drinking pattern and the risk of proteinuria but attenuated it, hypertension may partly explain this association. Further research on this association is needed.

Our study had several limitations. First, all of the subjects were middle-aged Japanese men who were from a single ethnic group and were employees of the same company. Thus, our results may have underestimated the associations in the general population because of the “healthy worker effect”; it is also unclear whether our findings apply to women, younger or older men, and other ethnic groups. Second, proteinuria was measured using a dipstick, but dipstick tests are more likely to yield false-positive and false-negative results than specific laboratory methods. However, dipstick tests are convenient and easy to perform in clinical practice and large epidemiological studies. Third, we did not obtain information about the types of alcoholic drinks. Finally, the proportion of current smokers was high at baseline (57%; see Table 1). However, it was not higher than that among the general Japanese male population in 2000, when we started this cohort study, because the 2000 Japanese National Health and Nutrition Survey reported that 54%–55% of Japanese men in their forties and fifties were current smokers.^29^

In conclusion, middle-aged Japanese men with an alcohol consumption of 0.1–23.0 g ethanol/drinking day on 4–7 days per week had a lower risk of consecutive proteinuria compared with non-drinkers. In contrast, men with an alcohol consumption of ≥69.1 g ethanol/drinking day on 4–7 days per week had an increased risk of developing consecutive proteinuria. Further research into the mechanism of the association between alcohol consumption and proteinuria is necessary.

## ONLINE ONLY MATERIAL

Abstract in Japanese.
