# Supplementary material for: Relationship Between Alcohol Drinking Pattern and Risk of Proteinuria: The Kansai Healthcare Study
Source: J Epidemiol. 2016 Sep 5;26(9):464–70. doi: 10.2188/jea.JE20150158 (PMC5008966; doi:10.2188/jea.JE20150158)
Supplement: Abstract in Japanese. [file je-26-464-s001.pdf]

Relationship Between Alcohol Drinking Pattern and Risk of Proteinuria: The Kansai Healthcare Study

飲酒パターンと蛋白尿発症リスクの関係: 関西ヘルスケアスタディ

上原　新一郎[1]、林　朝茂[1]、佐藤（粉河）恭子[1]、衣畑　成紀[1]、柴田　幹子[1]、大上　圭子[2]、神戸　泰[2]、橋本　久仁彦[2]

[1] 大阪市立大学大学院医学研究科　産業医学

[2] 西日本電信電話株式会社　関西健康管理センタ

【背景】適度な飲酒は心血管疾患、代謝疾患の発症リスクを低下させるという報告がある。飲酒パターンが蛋白尿の発症リスクとの関係があるかどうかについては不明である。

【方法】対象は、糖尿病のない、推算糸球体濾過量が 60 mL/min/1.73 m^2 以上で、蛋白尿を認めず、高血圧の治療を受けていない、40–55 歳の日本人男性 9154 人である。問診票によりアルコール週当たりの飲酒日数と飲酒日のアルコール摂取量を得た。発症は、毎年の試験紙法による尿検査で 1+以上を蛋白尿とし 2 年連続認めたものを「連続蛋白尿」と定義した。

【結果】81147 人年の経過で 385 人の連続蛋白尿の発症を認めた。多変量解析の結果、週当たりの飲酒日が 4–7 日で飲酒日に 0.1–23.0 g エタノール摂取者は、非飲酒者と比べて、連続蛋白尿発症リスクの低下を認めた (多変量補正後のハザード比 0.54 (95%信頼区間 0.36–0.80)）。一方で同頻度で飲酒日に 69.1 g エタノール以上の摂取者は有意に連続蛋白尿発症リスクが高かった (1.78 (1.01–3.14))。飲酒日が 1-3 日に 0.1–23.0 g エタノール摂取者は連続蛋白尿の発症リスクの低下を認めた（0.76（0.51-1.12））。また、同頻度で飲酒日に 69.1 g エタノール以上の摂取者は連続蛋白尿発症リスクが高かった (1.58（0.72–3.46）)が統計学的な有意差を認めなかった。

【結論】飲酒日のアルコール摂取量が 0.1–23.0 g エタノールの頻回飲酒者は最も蛋白尿の発症リスクが低かった。一方、頻回の 69.1 g エタノール以上の摂取者は蛋白尿の発症リスクが高かった。

キーワード ; アルコール、飲酒パターン、蛋白尿、慢性腎臓病、前向きコホート研究
